# Intention to leave and associated factors among health professionals working at public hospitals in East Gojjam zone, Northwest Ethiopia, cross-sectional study

**DOI:** 10.1371/journal.pone.0301235

**Published:** 2024-03-25

**Authors:** Friehiwot Molla, Muluye Molla, Endalkachew Delle, Geta Asrade, Bekalu Endalew

**Affiliations:** 1 Department of Public Health, College of Medicine and Health Sciences, Debre Markos University, Debre Markos, Ethiopia; 2 Department of Health System and Policy, Institute of Public Health, College of Medicine and Health Sciences, University of Gondar, Gondar, Ethiopia; Drexel University, UNITED STATES

## Abstract

**Background:**

The most important element of health systems for meeting the population’s healthcare demands is the workforce. The main issue facing the health industry, particularly in emerging nations, has been their shortage and turnover. Thus, the purpose of this study was to assess the intention of leaving and related variables among medical professionals employed at East Gojjam zone public health hospitals.

**Methods:**

An institution based cross-sectional study was conducted among 561 randomly selected health professionals working at public hospitals in East Gojjam Zone from 04 March 2019 to 30 March 2019. Self-administered structured questionnaire was used for data collection. Both bi-variable and multivariable logistic regression analysis were fitted. Variables having P-value less than 0.2 during bi-variable regressional analysis were entered in to multivariable logistic regression analysis and Adjusted Odds Ratio (AOR) with 95% confidence interval (CI) was used to declare the associated factors with intention to leave.

**Results:**

Overall, 61.3% (95% CI: 57.2, 65.4) of health professionals were intended to leave their working organizations. Dissatisfaction with work nature (AOR: 3.01; 95% CI: 2.05, 4.43), work environment (AOR: 1.83, 95% CI: 1.25, 2.68), Remuneration (AOR: 1.89; 95% CI: 1.29, 2.76), having low normative commitment (AOR: 0.55; 95% CI: 0.38, 0.81) and being unmarried (AOR: 1.78; 95% CI: 1.23, 2.58) were satistically significant factors with intention to leave their working organizations.

**Conclusions:**

The health professionals’ intention to leave their working organizations was high, three-fifth of the health professionals had intention to leave their organization which might result great service quality compromization and decrease the responsiveness of the health institutions in the study area. Dissatisfaction with remuneration, working environment, work nature, low normative commitment and being unmarried were factors associated with health professional’s intention to leave their working organizations. Therefore, hospital administrators, supervisors, and Healthcare policymakers need to emphasize on retention of health workers at their working organization by taking into account the above significant variables. Such as, through creating an attractive working environment and designing better benefit mechanisms.

## Introduction

Health professionals are the most vital component of health systems. They are central to attain, sustain and accelerate progress on universal health coverage [1,2]. The availability of a suitable number of health professionals determines the performance and efficacy of the health system in meeting diverse community health requirements and health-related goals [3,4]. The shortage of health professionals and issues with retention have been important issues in the health sector, particularly in developing countries. Professional turnover is a major contributor to the shortage of health professionals [5].

Employees’ projected likelihood of quitting their current job or finding another in the near future is referred to as "intention to depart," and it is used as a proxy for actual leaving [6].

Employees’ intentions to leave and health-care facility instability are significant globally, especially in poor countries[7,8]. In Africa, the percentage of people planning to depart varies between 18.8% and 41.4 percent [9]. Even though the number of skilled health professionals in Ethiopia is increasing, the gains are being offset by annual losses as many health professionals leave their jobs[10]. The national ratio of health professionals per 1000 population is 0.8, which is lower than the WHO’s recommended threshold of 2.5 health workers per 1000 population [11–13]. In Southwest Ethiopia, 59.4 percent of health workers planned to leave their jobs [14]. Similarly, in Amhara National Regional State, Ethiopia, 60.2% of nurses had the desire to leave their current working place [15]. Previous studies revealed that job satisfaction, types of profession (being physician), work experience, satisfaction to management system and salary were contributing factors for intention to leave [14].

The intention of health professionals to leave has a substantial impact on the operation of the healthcare sector around the world, particularly in developing countries, and obstructs progress toward health and health-related development goals [16]. The intention to quit leads to actual departures, a current shortage, and misallocations of health professionals. It also restricts community access to high-quality health care, particularly in rural areas where the poor and vulnerable are concentrated [17].

The intention of a health professional to leave their organization results in a large cost to the organization, as well as a negative impact on job quality in the form of withdrawal, diminishing involvement, absenteeism, avoidance behavior, and lower performance [2,18–20]. Thus, identifying factors that push health professionals to leave their working organization is important in order to enable the planners to increase retention, enhance performance, productivity and to promote a safe healthcare system. Analyzing the intention of a health professional to quit their current employer is critical for health institution management to improve retention strategies, improve the healthcare system’s performance, and find opportunities for improvement. However, there is limited evidence regarding intention to leave and leading factors of health professionals working in public hospitals in Ethiopia, particularily in the study area. Therefore, this study was aimed to assess intention to leave and associated factors among health professionals working at East Gojjam Zone public hospitals, Northwest Ethiopia.

## Methods and materials

### Study design, setting and period

An institution based cross-sectional study design was conducted among health professionals working at East Gojjam Zone public hospitals from 04 March 2019 to 30 March 2019. East Gojjam zone is one among the eleven administrative zones of Amhara National Regional State (ANRS), Ethiopia. Based on 2007 census conducted by the central statistics agency (CSA) of Ethiopia, East Gojjam zone has projected total population of 2, 663,005, of which 1,320,930 were males [21]. It has 21 districts, nine primary hospitals, one general hospital, 102 health centers and 406 health posts. It has a total of 2,905 healthcare providers. Among these healthcare providers, 1,129 were working at ten public hospitals, 1,776 were working at health centers and 812 were working at health posts.

### Study population

All health professionals who were working for at least six months and above at randomly selected public hospitals of East Gojjam Zone were included in this study while health professionals who were giving voluntary services and temporarily employed were excluded from this study.

### Sample size and sampling procedures

The sample size was determined using a single population proportion formula by assuming the proportion of health professionals’ intention to leave 59.4%, from a study done in southwest Ethiopia health institutions [14], 95% confidence level, 5% margin of error, 1.5design effect and 5% non-response rate, n = $({Za/2)}^{2}(P)(1-P)/{\left(d\right)}^{2}$, where n is sample size, p is proportion of health professionals intended to leave,1-p is proportion of health professionals not intended to leave and d is margin of error. The final sample size was 583.

**Fig 1:**
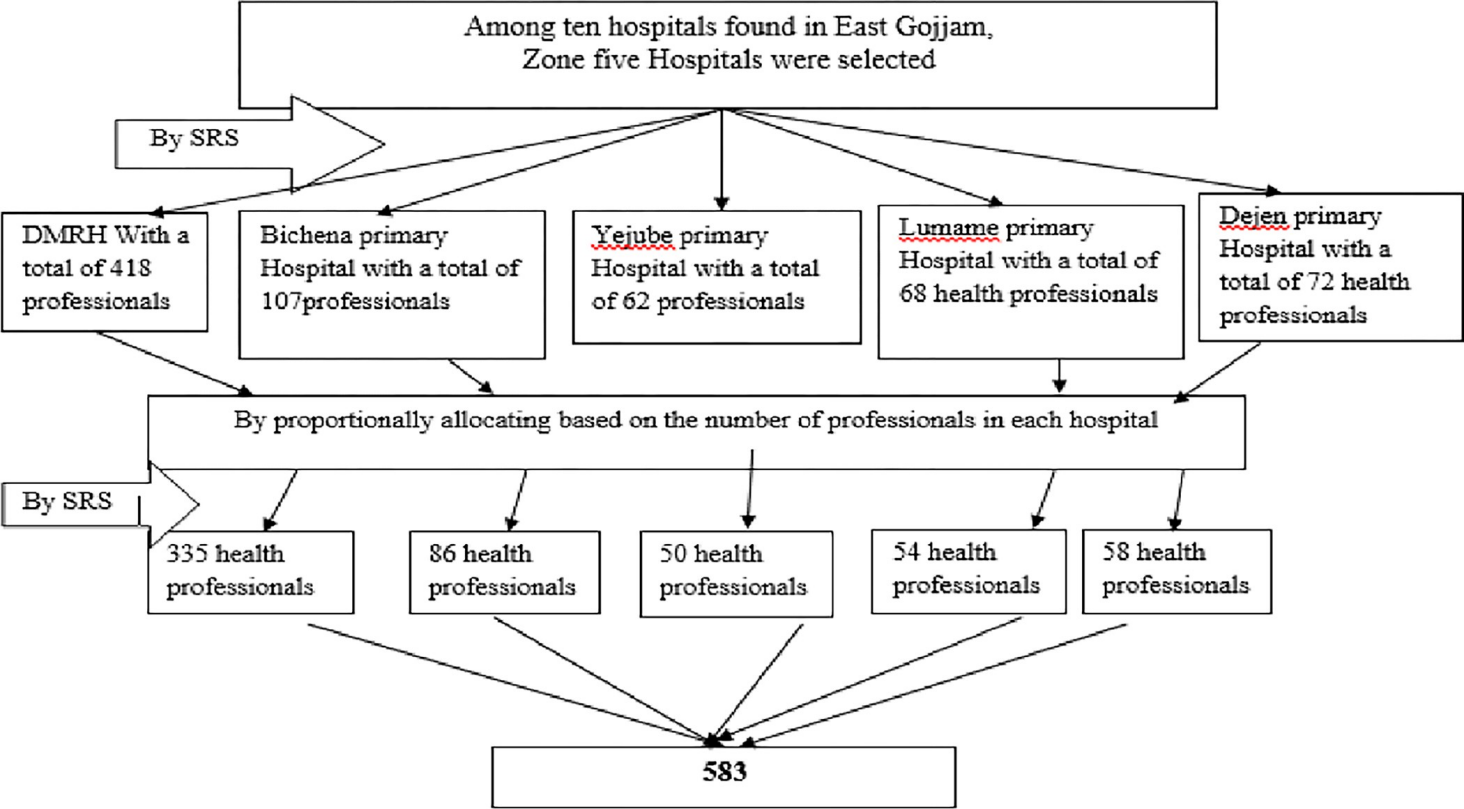
Schematic presentation of sampling procedure for intention to leave and associated factors among health professionals working in east Gojjam zone public hospitals, Northwest Ethiopia.

The study participants were chosen using a multi-stage simple random sampling technique. First, five hospitals (50%) were chosen by lottery method from a total of 10 hospitals in the East Gojjam Zone. Then, based on the number of professionals at each hospital, proportional allocation of a sample was done. Finally, by using hospitals’ payroll registration as a sampling frame simple random sampling technique was utilized and study participants were recruited from each profession proportionally (Fig 1).

### Measurements and variables

Intention to leave was the study’s outcome variable. Socio-demographic characteristics (age, sex, residence, educational status, marital status, types of profession, work experience, having children,distance from family and income level), Organizational commitment factors (normative commitment, continual commitment and affective commitment), job related factors (job reward, work load, work nature, work environment, remuneration (incentives), supervision, autonomy, peer-group relationship, organizational policy, management system and organization appraisal system) were the study’s independent variables.

A three-item Likert scale questionnaire was used to assess the intention to leave, with responses ranging from (1) strongly disagree, (2) disagree, 3(neutral), (4) agree, and (5) strongly agree. If they scored above the mean value, study participants were classified as intending to leave wehereas if they scored below the mean value, they were classified as not intending to leave [22–24].

Job satisfaction was measured using 37 Likert scale questions across six domains (work nature = 4 items, work environment = 9 items, remuneration and benefits = 12 items, coworker relationships = 4 items, autonomy = 3 items and supervision = 5 items). Participants were classified as satisified if they scored above the mean value and disatisified if they scored less than or equal to the mean value [25,26].

Organizational commitment was measured using affective (4 items), normative (3items) and continual (4 items) questions. Participants who scored above mean value for each components were considered as having high affective, normative and continuance commitments respectively [27].

### Data collection tools and procedure

Data were collected using pre-tested structured self-administered questionnaire by hand-delivering questionnaires to respondants and return later (to decrease respondent bias related to unclarified question), which was adapted from different literatures [24,28–30]. Five-diploma and two BSC nurses were recruited as data collectors and supervisors respectively.

### Data quality control

Data collectors and supervisors received one-day training on the study objectives, data collection instruments, methodologies, and producers prior to data collection. A pretest was conducted on 30 (5%) health professionals in Motta primary hospital, which was outside the study area, and necessary changes were made based on the results of the pre-test. The lead investigator and supervisors reviewed the consistency and completeness of the data on a daily basis. Cronbach’s alpha was used to assess the internal consistency of each dimension of the questionnaire. Each dimension has a Cronbach’s alpha more than 0.7, with the outcome variable having a Cronbach’s alpha of (= 0.81).

### Data processing and analysis

Data were entered, coded and cleaned in to Epi-data version 3.1 and exported to SPSS version 20 software for analysis. Both descriptive and inferential statistics were computed and results were presented using texts, tables, and figures. Both bi-variable and multi-variable logistic regression analysis was done to identify associated factors with intention to leave. Variables with p-value of less than 0.2 in the bi-variable logistic regression analysis were entered to multi-variable logistic regression analysis to filter out confounding factors. In multivariable logistic regression analysis variables with p-value of less than 0.05 and AOR with 95% CI were considered significantly associated factors of the outcome variable. Model fitness was checked using the Hosmer–Lemeshow goodness-of-a-fit test (p = 0.11) and Multicolinearity was cheked using variance inflation factor (VIF). VIF < 10 and tolerance greater than 0.1 were used to declare the absence of multi-collinearity.

### Ethical consideration

Ethical clearance was obtained from the Ethical Review Committee of the University of Gondar, College of Medicine and Health Sciences from Research and Publication Directorate office (Ref. No. IPH/180/06/2019). A formal letter was obtained from East Gojjam Zonal Health Department and the respective hospital administrators. The participants were then fully briefed about the study’s purpose and benefits and obtained informed written consent for both data collection and publication. Confidentiality was maintained through anonymity and privacy measures were taken to preserve the right of the participants throughout the research work including publication. Finally, the selected participants were asked about their willingness to join the study. Any study participant willing to engage in the study and those who wanted to stop an interview at any time were allowed to do so. This study was conducted in accordance with the Declaration of Helsinki.

## Results

### Socio-demographic characteristics of the respondents

A total of 561 study participants were participated in the study with a response rate of 96%. The mean age of the participants was 29.16 (±5.492 SD) years. Majority of the respondents 347(61.9%) were males. More than three-fifth (72.02%) of the respondents were degree and above holders and about two-fifth (39.9%) respondents had greater than 5 years’ working experience. Moreover, one hundred ninety-six (34.9%) respondents had children and majority of respondents 335 (59.7%) were working far from their parents (Table 1).

**Table 1:** Socio-demographic characteristics among health professionals working at public hospitals in East Gojjam Zone Northwest Ethiopia, 2019 (n = 561).

Variables	Category	Frequency	Percentage (%)
Age (years)	21–30	400	71.3
	31–40	134	23.9
	41–50	27	4.8
Sex	Male	347	61.9
	Female	214	38.1
Marital status	Unmarried	296	52.76
	Married	265	47.24
Religion	orthodox Christian	551	98.2
	Muslim	7	1.2
	Protestant	3	0.5
Educational status	Diploma	157	27.98
	Degree and above	404	72.02
Field of study (profession)	Nurse	288	51.3
	Medical doctor	74	13.2
	Pharmacy	66	11.8
	Midwifery	60	10.7
	Laboratory	49	8.7
	Radiology	12	2.1
	Health officer	9	1.6
	Others*	3	0.5
Work experience	< 2 years	160	28.5
	2–5 years	177	31.6
	> 5 years	224	39.9
Distance from parents	Far	335	59.8
	Near	226	40.2
Live With their children	Yes	196	34.9
	No	365	65.1
Monthly income in ETB (1USD = 28.8ETB)	≤4500	214	38.1
	4501–6500	224	39.9
	≥6501	123	21.9

ETB: Ethiopian Birr

* others (emergency surgeon, physiotherapy and sanitary).

### Intention to leave, organizational commitment and job satisfaction

**Fig 2:**
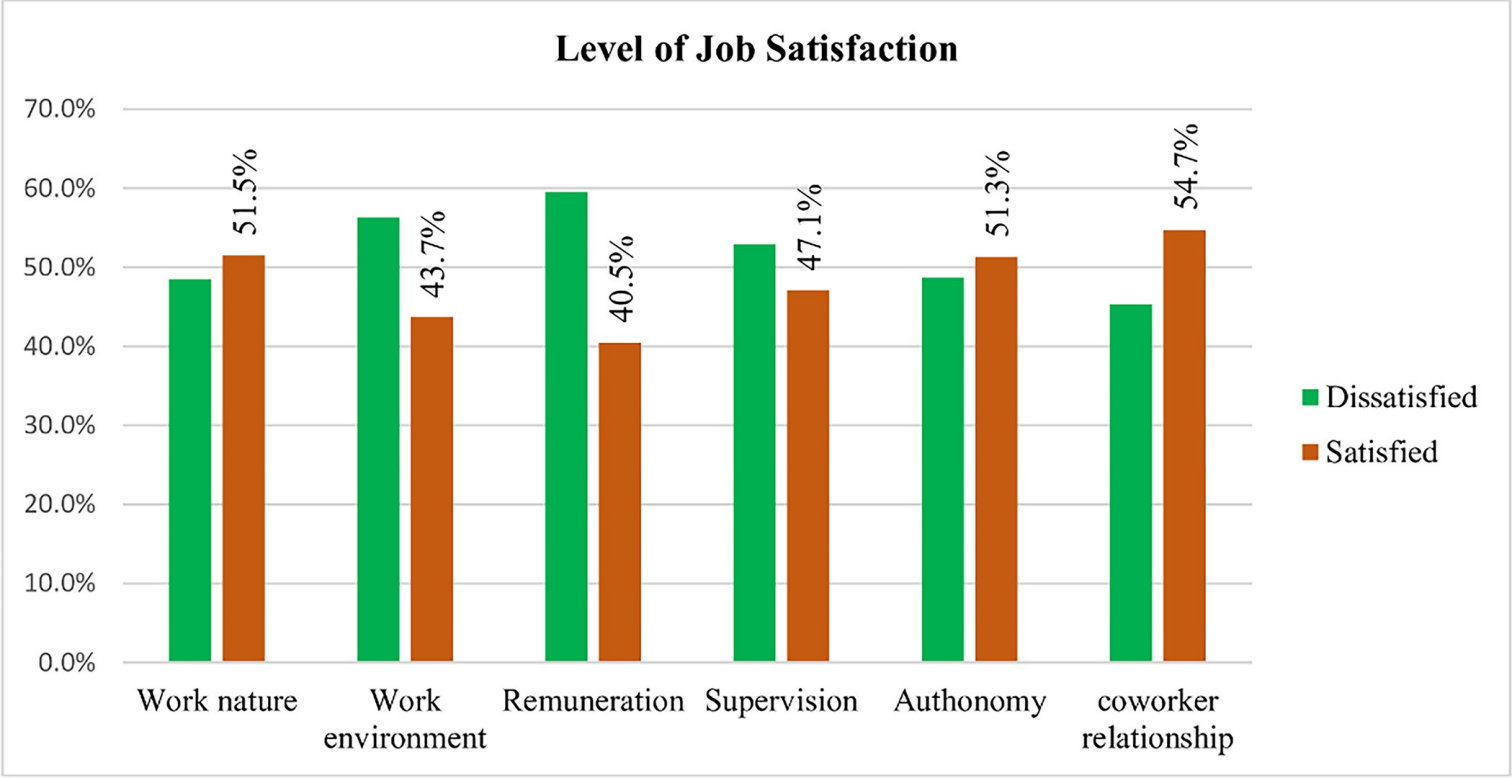
Factors associated with job satisfaction among health professionals working at public hospitals in East Gojjam zone North West Ethiopia, 2019.

Overall, 61.3% (95% CI: 57.2, 65.4) of study participants had an intention to leave their working place. About 55% of health professionals were satisfied with coworker relationship. On the other hand, majority of study participants (60%) were dissatisfied with remuneration (benefit packages) and the working environment (56%) (Fig 2).

**Fig 3:**
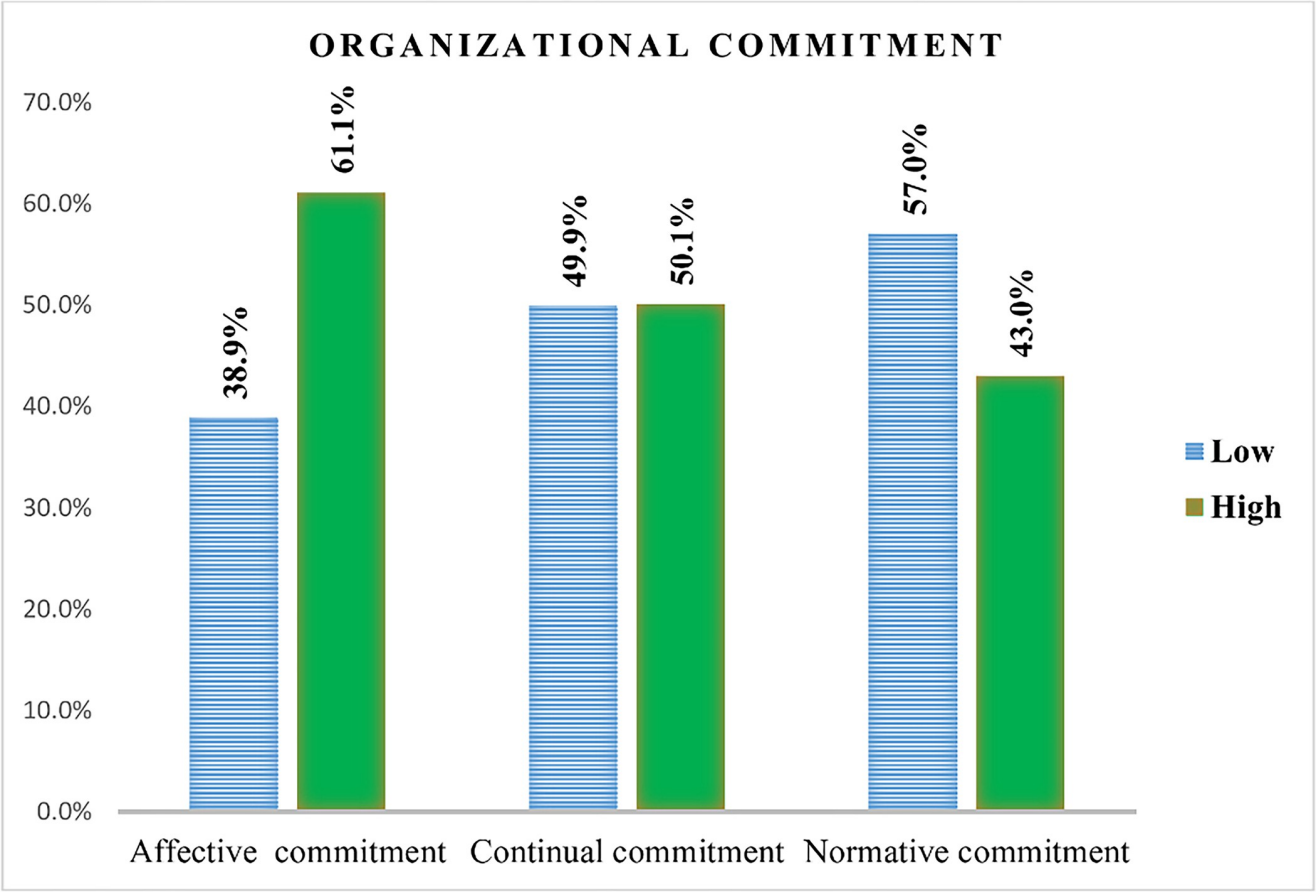
Organizational commitment factors among health professionals working at public hospitals in East Gojjam zone North West Ethiopia, 2019.

Regarding organizational commitment aspect, the majority of the respondents 343 (61.1%) had high affective commitment and low level (57%) of normative commitment (Fig 3).

### Factors associated with intention to leave

The multivariable logistic regression analysis illustrates that variables such as the status of satisfaction with work nature, work environment, remuneration (benefits), normative commitment and marital status remained to be significantly associated with intention to leave their working organization in the study area.

Accordingly, the odds of intention to leave their working organization were 1.78 times higher in health professionals who were unmarried than married (AOR: 1.78; 95% CI: 1.23, 2.58). Health professionals who were dissatisfied with the work nature were three times(AOR: 3.01; 95% CI: 2.05, 4.43) more likely to leave their working organization than those who were satisfied with the work nature.

The odds of intention to leave their working organization were 1.9 times higher in health professionals who were dissatisfied with remenuration than their couter parts (AOR: 1.9; 95% CI: 1.29, 2.76).

In addition, health workers who were dissatisfied with their working environment were 1.8 times (AOR: 1.83 95% CI: 1.25, 2.68) more likely to leave their working organization than their counterparts. Moreover, health professionals having a low level of normative commitment were 55% less likely (AOR: 0.55; 95% CI: 0.38, 0.81) to leave their working organization as compared with those health professionals with a high level of normative commitment (Table 2).

**Table 2:** Bi-variable and multi-variable logistic regression analysis of factors associated with intention to leave among health professionals working at public hospitals in East Gojjam zone North West Ethiopia, 2019 (n = 561).

Variables	Category	Intention to leave		Odds Ratio	
		Yes	No	COR (95%CI)	AOR (95%CI)
Marital status	Unmarried	198	98	1.6 (1.17, 2.32)	1.78(1.23, 2.58)*
	Married	146	119	1	1
Distance from parents	Far	216	119	1.39(0.98, 1.96)	1.20 (0 .81, 1.78)
	Near	128	98	1	1
Educational status	Diploma	89	68	1	1
	Degree and above	255	149	1.31(0.99,1.90)	0.77(0.51,1.61)
Monthly Salary (ETB)	≤4500	130	84	1.21(0.77,1.99)	1.33(0.75, 2.38)
	4501–6500	145	79	1.44(0.92,2.25)	1.47(0.89, 2.42)
	≥6501	69	54	1	1
Supervision	Dissatisfied	190	107	1.27(0.90, 1.78)	0.81(0.53,1.24)
	Satisfied	154	110	1	1
Autonomy	Dissatisfied	176	97	1.29(0.92,1.82)	0.92(0.62, 1.38)
	Satisfied	168	120	1	1
Work nature	Dissatisfied	204	68	3.19(2.23, 4.57)	3.01(2.05,4.43)*
	Satisfied	140	149	1	1
Work environment	Dissatisfied	222	94	2.38(1.68, 3.37)	1.83(1.25, 2.67)*
	Satisfied	122	123	1	1
Remuneration	Dissatisfied	232	102	2.34(1.65,3.31)	1.88(1.29, 2.76)*
	Satisfied	112	115	1	1
Normative commitment	Low	184	136	0.68(0.48, 0.96)	0.55 (0.37, .81)*
	High	160	81	1	1
Affective commitment	Low	150	68	1.69(1.18, 2.42)	1.11(.72, 1.69)
	High	194	149	1	1
Continual commitment	Low	184	96	1.45(1.03, 2.04)	1.42(.98, 2.07)
	High	160	121	1	1

AOR: Adjusted Odds Ratio; CI: Confidence Interval; COR: Crude Odds Ratio; ETB: Ethiopian Birr

* P<0.05, 1: Reference category.

## Discussion

The aim of this study was to examine the magnitude of health professionals’ intention to leave from their working organization and its determinant factors. Overall, 61.3% (95% CI: 57.6, 65.6) of health professionals who were currently working at public hospitals of East Gojjam zone had an intention to leave their working organizations. This means that only 38.7% of health professionals work to enhance the health system and live in a stable environment, which is insufficient to accomplish the health system’s ultimate goals of responsiveness, financial risk reduction, and improved health. This finding was in line with studies done in North Shoa zone Ethiopia (61.3%) [23], Horo Guduru Wollega zone, Northwest Ethiopia (65%) [8], Jimma zone public health centers: Southwest Ethiopia (63.7%) [24], a study finding conducted in southwest Ethiopia health institutions(59.4%) [14] and Turkey (60.9%) [31].

However, the finding of this study was lower than the studies conducted in Yirgalem and Hawasa referral hospitals southern Ethiopia (83.7%) [32], Hubei, China (78.35%) [20] and Ghana (69%) [33]. The possible explanation might be due to the difference in the study participants and study settings. For instance, the study in china includes only general practitioners in which general practitioners have more alternatives employing organization relative to other health professionals and the study in Ghana considers both health center and hospitals, unlike this study.

On the other hand, this result was higher than a study done at university of Gondar specialized hospital (52.5%) [28] and Sidama zone public health facilities in South Ethiopia(50%) [34]. This variation could be due to the difference in the study setting and period. This study was considered both primary and referral hospitals [35]whereas the study conducted at Gondar included health professionals only from the referral hospital.

The results of this study showed that unmarried health professionals were more likely to leave their current working organization than those who were married. This is consistent with studies conducted in Northwest Ethiopia referral hospitals on the Nursing profession [15], in Saudi Arabia [26], and in Turkey [31]. This finding could be explained by being single have fewer family responsibilities, thus making them more mobile. On the other hand, most married people had more dependent families, which could create a preference for stability [35].

Health professionals who were dissatisfied with work nature were more likely to leave their working hospital than those who were satisfied. This finding was appeared to be consistent with a study conducted in north shoa zone, northwest Ethiopia [23]. This finding was also supported by Herzberg two factor theory of motivation. This theory identified factors like recognition, work condition, nature of work, responsibility, and advancement impacts jab satisfaction, which in turn influences employee’s intention to stay or leave their working organization [36]. The possible explanations might be if healthcare workers who distaste the activities they perform at work and feel a deficiency in their personal accomplishments might be initiated to leave from their organization.

The other job satisfaction factor, remuneration (benefit packages) was among the significant predictors in which health professionals who were dissatisfied with remuneration and other benefit package were more likely to leave their current working hospital compared to their counterparts. This finding was supported by study findings conducted in Ethiopia, North Shoa zone [23] and Horo Guduru Wollega zone [8]. This could be explained in that if health workers perceived there is an inadequate and incomparable benefit for the task they perform, they may prefer to leave. Whereas satisfied health professionals want to remain within the organization because of their need to maintain the benefits they received.

Health workers who were dissatisfied with the work environment were more likely to leave than those who were satisfied with it. This finding was in agreement with studies done at public health facilities in Horo- Guduru Wollega zone southwest Ethiopia [8], in Sidama zone southwest Ethiopia [34] in Ghana [34], and a study conducted in Chinese [7]. This might be explained by if working conditions are substandard or the workplace lacks important facilities such as proper lighting, furniture, restrooms, and other health and safety provisions, employees will not be willing to put up with the inconvenience for a long time.

The other significant determinant factors were organizational commitment. Health workers who had low normative commitment were less likely intended to leave their current working organization as compared with those health workers who had a high normative commitment by 45%. This finding is in contrary to studies in North Shoa zone northwest Ethiopia [23], East Gojjam zone North West Ethiopia [29] and Northwest Ethiopia on Nursing profession [15]. This variation might be due to health professionals who had low normative organizational commitment may enhance a feeling of responsibility, honesty, and autonomy, which may lead to job satisfaction so less intent to leave than those who had high-level normative commitment.

### Limitation of the study

Use of self-reporting measures may dispose of reporting bias, because of the respondents’ interpretation of the questions. Furthermore, this study was not supplemented with a qualitative method to support the quantitative findings. The other limitation was the lack of follow-up, in which the researcher could compare participants’ intentions to leave or stay with their actual turnover actions.

## Conclusion

The health professionals’ intention to leave their working organizations was high, three-fifth of the health professionals had intention to leave their organization which might result great service quality compromization and decrease the responsiveness of the health institutions in the study area. Dissatisfaction with remuneration, working environment, work nature, low normative commitment and being unmarried were the factors statistically significant that affect health professional’s intention to leave their working organization at public hospitals in East Gojjam Zone Northwest Ethiopia. Therefore, hospital administrators, supervisors, and Healthcare policymakers need to emphasize on retention of health workers at their working organization by working on their remuneration, creating conducieve environment and designing better benefits mechanisms.
